# A Comprehensive Update of Cerebral Organoids between Applications and Challenges

**DOI:** 10.1155/2022/7264649

**Published:** 2022-12-05

**Authors:** Xiaodong Li, Abdullah Shopit, Jingmin Wang

**Affiliations:** ^1^Department of Obstetrics and Gynecology, The First Affiliated Hospital of Dalian Medical University, Dalian 116044, China; ^2^Academic Integrated Medicine & Collage of Pharmacy, Department of Pharmacology, Dalian Medical University, Dalian 116044, China

## Abstract

The basic technology of stem cells has been developed and created organoids, which have established a strong interest in regenerative medicine. Different cell types have been used to generate cerebral organoids, which include interneurons and oligodendrocytes (OLs). OLs are fundamental for brain development. Abundant studies have displayed that brain organoids can recapitulate fundamental and vital features of the human brain, such as cellular regulation and distribution, neuronal networks, electrical activities, and physiological structure. The organoids contain essential ventral brain domains and functional cortical interneurons, which are similar to the developing cortex and medial ganglionic eminence (MGE). So, brain organoids have provided a singular model to study and investigate neurological disorder mechanisms and therapeutics. Furthermore, the blood brain barrier (BBB) organoids modeling contributes to accelerate therapeutic discovery for the treatment of several neuropathologies. In this review, we summarized the advances of the brain organoids applications to investigate neurological disorder mechanisms such as neurodevelopmental and neurodegenerative disorders, mental disorders, brain cancer, and cerebral viral infections. We discussed brain organoids' therapeutic application as a potential therapeutic unique method and highlighted in detail the challenges and hurdles of organoid models.

## 1. Introduction

The human induced pluripotent stem cells (hiPSCs) and human embryonic stem cells (hESCs) have rapid and advanced progress, which provided new insights for research of neurological diseases and human brain development [1]. The three-dimensional (3D) organoids have been created through the development of the stem cells' basic technology, which established a strong interest in regenerative medicine. Cerebral organoids are used to simulate different human brain regions, which reproduce specific brain structures, including the cerebellum [2], midbrain [3, 4], hypothalamus [5], hippocampus [6], and pituitary gland [7]. The 3D models live for long periods, may more than 25 months, [8] which makes it a great and premium model to investigate brain development and cerebral disease mechanisms [9–13]. Recently, cutting-edge technologies including single-cell sequencing and gene-editing advances have been applied in 3D models, which has generated unmatched possibilities for neurological disease modeling. In this review, we highlight recent advances in the brain organoids and their applications as promising models for studying brain development and cerebral disease mechanisms. We then discuss the brain organoids' therapeutic application as a potential therapeutic unique method. Despite the enormous promise of applications of brain organoid models, we explain the current major challenges, hurdles, and limitations of organoid models used. Moreover, we display feasible and constructive suggestions for the future that would contribute to developing medical research.

## 2. Specialized Brain Organoids

Different types of cells with the development of technologies have been used to generate organoids, which include interneurons and oligodendrocytes (OLs) [14]. OLs are fundamental for brain development, including electrically sequestered neuronal axons and myelinating for impulse transmission, as well as metabolic support to neurons and provide nutrition. However, findings of single-cell sequencing reference that cortical organoids have a deficiency in the cells of the oligodendrocyte progenitor [8, 15]. Some studies have exposed advanced organoids to beat these issues by oligodendrocyte growth factors in cortical spheroids that were through inducing myelinating OLs and oligodendrocyte progenitors [16]. There is a protocol for the observation and acceleration maturation of OLs, nine weeks after organoid formation. Promote and improve myelination, oligodendrocyte production, and phenotypes of myelination defect diseases using promyelinating drugs have monitored production of OLs via OLIG2-green fluorescent protein (GFP) signal and create forebrain organoids by applying the GFP stem cell reporter line [17]. There is another protocol for organoid culture development, which produces neurons, astrocytes, and OLs. That protocol uses to study the myelination, development of OLs, and main cell types interaction in the central nervous system, which applies a set of growth factors and small molecules [18]. Interneurons play a central role in regulating the cortical network processes. Some studies have established organoids to recapitulate the human medial ganglionic eminence (MGE) development. These organoids contain essential ventral brain domains, neuronal networks, and functional cortical interneurons, which are similar to the developing cortex and MGE [19]. The enrichment of cerebellar disease genes has been investigated in distinct cell populations in the cerebellar organoids. That demonstrated xeno-free human cerebellar organoids as a unique valuable model to gain insight into cerebellar development and its associated disorders [20].

## 3. Applications of Brain Organoids

Numerous studies have displayed that brain organoids can recapitulate fundamental and vital features of the human brain, such as cellular regulation and distribution, neuronal networks, electrical activities, and physiological structure. So, brain organoids have provided a singular model to study and investigate neurological disorder mechanisms (Table 1).

## 4. Neurodegenerative Disorders

### 4.1. Alzheimer's Disease

The progressive worsening of physical functions, behavioral impairment, and cognitive decline are clear manifestations of Alzheimer's Disease (AD), which is the most common neurodegenerative disease. Some reports demonstrated that have created a 3D culture system by amyloid-*β* precursor protein (APP) and presenilin 1 (PSEN1) upregulated human neural stem cells; that observed clearly the pathological aggregation of amyloid-*β* and Tau, suggesting the 3D culture advantage. [47, 48]. There has been observed spontaneous and persistent aggregation of Ab in the neural organoids derived from patients with familial Down syndrome (fAD). A significantly high pTau immunoreactivity has been displayed in fAD organoids at the later stage of culture compared to the control group. The pathologic changes induced by Tau phosphorylation and amyloid b in fAD organoids have been reduced by inhibitors of beta and gamma-secretase [49]. For neurodegenerative diseases therapeutic compounds screening, the cerebral organoids have many characteristics and can be useful. A recent finding shows that can directly establish a new model of AD by infecting the herpes virus into 3D brain-like tissues, which can simulate the formation of deteriorated functionality in the AD pathological process, neuroinflammation, gliosis, and amyloid plaques [50].

### 4.2. Parkinson's Disease

Parkinson's disease (PD) is the most second common neurodegenerative disease. Dopaminergic neuron impairment in the substantia nigra is the main characteristic of the PD; that typical motor symptoms include gait and postural disorders, resting tremors, muscle stiffness, and bradykinesia. Presently, the animal and cellular models of PD have some restrictions to mimic the PD phenotypes, such as animals with genetic mutations like mutations of LRRK2 cannot display clear progressive evidence of the Lewy body formation or loss of midbrain dopamine neurons [51, 52]. Organoids of midbrain specific derived from patients' sporadic PD with LRRK2-G2019S mutation consist of midbrain dopaminergic neurons (mDAN), but LRRK2 organoids have less in the mDAN complexity and number compared with the control group, which is harmonious with the PD patients' phenotype [53]. The heterozygous LRRK2-G2019S point mutation has been inserted into hiPSC leading to the isogenic midbrain organoids (MOs) created, using the technology of CRISPR-Cas9 [51]. The findings were that the corresponding markers including DAT, VMAT2, AADC, and TH expression were inhibited and shortened the dopaminergic neurons' neurite length in the mutant MO [51]. Besides, there are some pathological signs PD-related found in MOs also such as abnormal clearance of *α*-synuclein and increased aggregation. The gene expression profiling findings demonstrated there are many similarities between a PD patient's brain tissue and the mutant MOs. In mutant, MOs, specifically, were TXNIP overexpression, and the TXNIP suppression can inhibit the MOs phenotype induced by LRRK2, so maybe LRRK2-related sporadic PD has a correlation with TXNIP [51]. All these results exposed valuable pathophysiology insights for the progression and treatment of PD. Moreover, there is an early alteration in LIM homeobox transcription factor-alpha expression and late alteration in tyrosine hydroxylase markers in the MOs derived from idiopathic PD patients. In the forms of PD idiopathic, many related key genes have been determined such as FOXA2, LMX1A, PTX3, and neuronal marker genes TH [54]. Lately, it was reported that midbrain-like organoids, new type, have been developed, which can produce mDANs and have homogeneous and stable structures, glial cells, and other neuronal subtypes [55]. These findings indicate that MOs could be unique models for familiar and sporadic PD.

### 4.3. Ataxia-Telangiectasia

Ataxia-telangiectasia (A-T) is a genetic disorder caused by the lack of functional ATM kinase, which is characterized by neurodegeneration, neuronal defects, premature aging features, and chronic inflammation [56]. The association relationship between the neurological deficiencies of A-T and the detrimental inflammatory signature remains unclear [57]. Mechanistically, the cGAS-STING pathway is required for induction of a senescence-associated secretory phenotype (SASP) and the recognition of micronuclei in brain organoids. Furthermore, there was demonstrating that cGAS and STING suppression effectively inhibits astrocyte senescence and neurodegeneration, inhibits self-DNA-triggered SASP expression in A-T brain organoids, and ameliorates A-T neuropathology in the brain organoids [58].

### 4.4. Brain Cancer

Medulloblastoma (MB) is one of the most aggressive malignant brain tumors in children, which predominantly occurs in the cerebellum and has a high mortality rate [59]. The most aggressive subgroup of MB is group 3, which has c-MYC overexpression. The reports demonstrated that OTX2/c-MYC is a new driving gene wanted for 3 MB tumorigenesis in the cerebellar organoid of 3 MB. OTX2/c-MYC tumorigenesis in the organoids has been inhibited by treatment using EZH2 inhibitor tazemetostat [60]. Therefore, organoids of the human brain can be effective models applied to investigate the genetic mechanisms roles and treatment in glioma patients. Glioblastoma (GBM) accounts for 54% of all gliomas and is considered the most malignant type of brain cancer [61]. There were cerebral organoids used in vitro study primary human GBM model. The glioma cerebral organoids (GLICO) model has been obtained after the coculture of glioma stem cells (GSCs) with organoids. GSCs cocultured with organoids display deeply infiltrated and metastasized to the organoids inner zones and proliferated in host tissues that generated tumors closely related to GBM patients' tissue [62], suggesting that the GLICO model represents well the malignant GBM characteristics.

## 5. Neurodevelopmental Disorders

### 5.1. Autism Spectrum Disorders

One of the neurodevelopmental disorders that affect behavior and communication is Autism spectrum disorder (ASD) which is caused by various pathogenic factors, such as environmental factors, epigenetic modifications, and genetic mutation. The preference differentiation toward GABAergic neurons has been demonstrated in the cortical organoids derived from patients with ASD; however, glutamatergic neurons have not been the alterations, resulting in the imbalance of GABA-Glutamate neurons, which resulting from the FOXG1 expression alteration [63]. A multiomics investigation on the iPSC-derived cortical organoids has demonstrated an epigenomic and transcriptomic pattern similar to isogeneic tissue of the fetal brain, particularly during 5 to 16 weeks of gestation [64]. Moreover, cell types of the forebrain organoids were similar to embryonic prefrontal cortical; RNA sequencing of the organoids at the transcriptional profiles has the highest correlations with multiple forebrain structures of fetal brain tissue from the BrainSpan transcriptome database [65]. Additionally, been exposed 49,640 active transcription factors essential for the specification of cortical neurons [64], and genes expressed differentially are strongly correlated with the Wnt/b-catenin signaling pathway [66]. The volume of cerebral organoids with RAB39B mutation was large compared with control and has shown excessive proliferation and impaired differentiation of NPCs. AKT-mTOR-PI3K signaling pathway activation has been induced by RAB39B downregulation, and the phenotypes rescue can be by AKT-mTOR-PI3K signaling inhibition [67]. That was consistent with the report results of Jong et al., which showed an excess in volume and thickening of the cortical organoids with CNTNAP2 mutation that was related to increases in total cell number due to increased neurogenesis and neural progenitor cells (NPCs) proliferation [65]. CHD8 is a gene related to ASD; it has been shown that CHD8 regulates other genes related to ASD, such as AUTS2 and TCF4. In the CHD8 mutant brain organoids derived from iPSCs, ASD includes macrocephaly-autism disorder, and the function lack of RAB39B mutation leads to epilepsy, ASD, and macrocephaly [67]. Some interesting facts are beginning to unfold using the organoids model, as a recent study revealed that expression of ASD genes, especially speech and language difficulty-related gene FOXP2 overexpressed in an autistic savant [68].

### 5.2. Lissencephaly

The most serious form of lissencephaly type 1 is Miller Dieker's syndrome (MDS) which is characterized by seizures, decreased brain size, mental retardation, and craniofacial deformities [36]. Cerebral organoids derived from patients with MDS show decreased vertical divisions and increased apoptosis [69]. Furthermore, observed the delaying of the outer radial glial cells- (oRGCs-) specific cytokinesis, cell autonomy, and defects of neurons radial migration. These results display the involvement of oRGCs defects mitotic in the human lissencephaly pathogenesis. The ventricular radial glial cells (vRGCs) in the organoids of the forebrain derived from patients with MDS also show a shift from symmetrical to asymmetrical cell division [36]. Furthermore, in MDS organoids, there were many changes that have been detected in the ventricular niche organization, including the irregular situation of retracted cells from the apical membrane and the vRGC tissues having low compactness [36]. Regulating the *β*-catenin/N-cadherin pathway can treat these phenotypes, suggesting that Wnt signaling plays a vital function in MDS.

### 5.3. Down Syndrome

Down syndrome (DS) is a genetic disorder that is the most common dementia form in people <50 years old and is the most common reason for learning difficulties [70]. Dividing the DS dementia-causing factors into two categories, neurodegenerative and neurodevelopmental disorder, an imbalance in inhibitory and excitatory neurotransmission contributes mainly to DS cognitive deficits. DS organoids produce a variety of SSTC GABAergic and CRC neurons and numerous OLIG2C NPCs [71]. There are some conflicts between the culture of 2D and 3D that were noted; different subtypes of neurons can be generated from OLIG2C NPCs in 3D culture, while 2D culture can only obtain CRC neurons [71]. These results indicate that OLIG2 could be a potential target for DS therapy. Some phenotypes of AD were observed in patients with DS. Reports have detected that organoids derived from familial AD (fAD) patients and DS patients spontaneously demonstrate Tau hyperphosphorylation and deposition of amyloid plaque, which were more significant in AD than in fAD [72]. Moreover, delayed onset of dementia was in around 30% of patients with DS, which underlying mechanism may be due to the BACE2 triplication [73]. Likewise, T21-hiPSC organoids have been protected from early AD-like amyloid plaque pathology by BACE2 trisomic level [74]. These findings suggest that BACE2 has a physiological role in inhibiting AD and can be a therapeutic target for AD.

### 5.4. Neonatal Hypoxic Injury

The most common cause of neonatal disability and death is neonatal hypoxic injury (NHI), in which survivors usually suffer from cognitive impairment, epilepsy, and cerebral palsy [75]. There was a study on the effects of oxygen with different concentrations on the NHI brain organoids which were established for the investigation. These results display that expressions of the genetic markers CLIP2, DCX1, and FOXG1 for migrated cortical neurons, glial cells, OLs, and forebrain were inhibited by hypoxia, which could be suppressed using minocycline. Furthermore, using minocycline has decreased apoptosis in brain organoids induced by hypoxia [76].

### 5.5. Periventricular Heterotopia

The neocortex evolution process in mammals is highly consistent that depends on the neuron's maturation, migration, and precise generation. Periventricular heterotopia is one of the most common malformations of cortical evolution and is closely related to FAT4 and DCHS1 [77]. The iPSCs construct, and cerebral organoids have been established using somatic cells with FAT4 or DCHS1 mutations in patients. The morphology of the NPCs processes in organoids derived from iPSCs of a healthy person manifests carefully, straight and arranged. However, processes neuronal often display a distorted and destroyed morphological in the organoids with FAT4 knockout or mutation [78].

### 5.6. Primary Microcephaly

The most cause of primary microcephaly is genetic, which is regulating the cilium caused by autosomal recessive mutations such as CENPJ, CPAP, MCPH1, ASPM, CDK5RAP2, and WDR62 that genes also regulate centrosomes assembly [79]. Recently, specific brain organoids for congenital microcephaly have been generated, which have WDR62, ASPM, CDK5RAP2, and CPAP mutations [15, 79, 80]. The primary microcephaly cerebral organoid model has been established. Truncation mutations of somatic cells with heterozygous have been reprogrammed from CDK5RAP2 to iPSCs. From iPSCs of patients created neuroepithelial tissue which was small compared with the control group, after being transferred to neural induction. The brain organoids which were established contain many neurons and few radial glial stem cells (RGs), signifying that the decrease of CDK5RAP2 leads to premature neural differentiation with progenitor cells losing [81]. CPAP mutation can cause microcephaly and Seckel's syndrome. From the Seckel syndrome patient with CPAP mutation, brain organoids have been derived that display premature neuronal differentiation and smaller size [82]. Moreover, there was a demonstrated increase in the length and number of cilium of the Seckel organoids in comparison with control, suggesting that cilium breakdown is delayed [82]. These results confirmed that CPAP has a negative regulation in the cilium length and indicate that cilium plays a vital role in the NPCs maintenance. The organoids which have been iPSCs-generated with WDR62 mutation exhibit premature NPCs differentiation, slowed the cilia lengthening and decomposition, and reduced proliferation and cell cycle progression. The study of the mechanism has demonstrated that WDR62 is correlated with CEP170 and enhances CEP170 to locate in the primary cilia matrix, where CEP170 decomposes cilium through the microtubule depolymerization factor KIF2A activation [79]. These results display novel insights into primary microcephaly pathogenesis. Microcephaly organoids with ASPM mutation display poor lamination and few vRGCs, neuroepithelial tissues, and outer RGCs. Have been noticed in the ASPM mutant, organoids decreased electrical activity and maturation, which confirms the correlation and role of ASPM mutations in congenital mental retardation in patients [80]. A recent study has been concerned with the investigation of exposed microcephaly-related NARS1 mutations and whole-exome sequencing in >5,000 neurodevelopmental disorder patients. The cortical brain organoids, patient derived with NARS1 mutation, have been created, whereas the results have displayed inhibiting cell cycle and proliferation of RGCs and smaller size [38].

### 5.7. Progressive Microcephaly

Another microcephaly is called secondary microcephaly, which causes by infection, external environment, and other factors. Zika virus (ZIKV) infection is one of the causes of secondary microcephaly that has been widely studied. The binding of ZIKV particles to cell membranes and localizing them in cellular vesicles and mitochondria lead to inhibition of the neurosphere formation and cell death [83]. Some studies have developed an organoid of the forebrain and infected it with ZIKV at various pregnancy stages. There was significantly increase in the lumen size of the ventricular structure after the exposure of ZIKV at organoids in the early stages (day 14), while significantly reduced VZ zone size and thickness [5]. That was very similar to the central ventricular dilatation of the fetus brain infected with ZIKV and its clinical phenotypes [84].

## 6. CNS Infectious Diseases

### 6.1. Cerebral Malaria

Cerebral malaria is one of the severe clinical manifestations, which is associated with serious neurological complications [85]. Hemolysis is one of the most malaria complications that lead produces a by-product called heme, which enhances iPSCs spontaneous differentiation and apoptosis and induces brain injury-related biomarkers changes in organoids, such that BDNF, CXCR3, and CXCL-10 expression increased, while ERBB4 expression decreased. Furthermore, neuroprotective impacts on heme-treated organoids have been shown by neuregulin-1 [86]. Hence, the model of brain organoids can be used to investigate the effects of hemolysis on fetal brain evolution.

### 6.2. Virus Infections

The brain organoids development has extremely contributed to neurotropic viruses' study promotion and provided alternative ZIKV infection models for 2D cell culture and animal models [87]. A recent report demonstrated that exposure of the brain organoids to enoxacin can avoid the microcephalic phenotype by preventing ZIKV infection. These findings revealed the RNAi-mediated antiviral immunity physiological significance in human brain development especially in the early stages, discovering new strategies to promote RNAi's resistance to decrease congenital viral infection in humans [34]. Besides, has been investigated ZIKV neurotoxicity to study its mechanism and possible efficacy in GBM as an oncolytic virus, the findings of GBM cortical organoids have shown that ZIKV preferentially targets GSCs, showing effective oncolytic impacts. The GBM organoids application in preclinical studies augments selective tumor targeting and may provide oncolytic virus therapeutic positive implications [88]. Recent reports indicated expression of the ACE2 that is functionally required for SARS-CoV-2 infection has been demonstrated in brain organoids. Furthermore, the SARS-CoV-2 infections in the brain organoids showed the relationship between neuroinvasion and ischemic infarcts, which displayed that the more susceptible regions to the viral invasion were ischemic infarct regions [89]. An organoid model to study the choroid plexus (ChP) has developed recently, which recapitulates the epithelial polarization of ChP cells to investigate the viral tropism of SARS-CoV-2 in various cells of the CNS. The organoids showed susceptibility to SARS-CoV-2 and rather efficient ChP infection, leading to transcriptional deregulation and cell death susceptibility of lipoprotein-producing cells [90]. Japanese encephalitis (JE) infection is still a challenging issue across the world which causes irreversible brain damage [91]. JEV infection impaired the development of organoids by targeting oRGCs and astrocytes and NPCs and induces cell death [92].

## 7. Mental Disorders

Schizophrenia is one of the most serious mental disorders with neurodevelopmental origins, molecular neuropathology, and complex environmental/genetic reasons. There is a challenge in observing the mental illness phenotypes in rodents due to the structural and functional differences of brain regions in comparison with a human being [93]. Organoids of the forebrain derived from schizophrenia DISC1 mutant patients display modification of RGCs proliferation. The NDEL1 and DISC1 correlation plays a vital role in neural stem cell maintenance during human forebrain development [94]. The WNT signaling pathway overactivation has been detected in the isogenic DISC1 mutant brain organoids. DISC1 organoids morphological examination shows an impaired proliferation and mixed structural morphology, which can be treated by WNT antagonism [95]. Brain organoids derived from schizophrenia iPSCs show reduced neuronal proliferation and development and decreased FGFR1 expression in cortical cells, conjugated with loss signaling of nFGFR. Cortical growth arrest similar to schizophrenia can be generated by antagonist PD173074 with FGFR1 knockdown in control organoids. Besides, this can decrease the developmental abnormalities in cortical neurons through FGFR1 activation [96]. A recent study suggests that the found multiple mechanisms of schizophrenia in brain organoids and these different mechanisms link up upon primordial brain developmental pathways such as growth factor support, survival, and neuronal differentiation, which may integrate to promote the intrinsic risk of schizophrenia [97].

## 8. Organoids Therapeutic Applications

The difference in species may indicate that the use of animals for therapeutic development, drug investigation, and disease modeling does not closely represent biological responses in humans. In addition, the traditional cell culture 2D may not exactly represent modeling human diseases. Thus, using organoid models to investigate pathological and regulatory molecular mechanisms is a promising strategic choice. Treatment of the organoids with standard therapy, chemoradiotherapy, displays as seen in practice through comparatively low response. Treatment of organoids showed general therapeutic resistance with apoptotic and antiproliferative effects differing biological mechanisms from those of 2D cultures [98]. The model of brain organoids can use to study some compounds for neurodevelopmental disorders, such as ZIKV antiviral drugs. The reported study investigates two potential drug compounds, amodiaquine dihydrochloride dehydrate, and hippeastrine hydrobromide, which could prevent ZIKV infection in cortical NPCs and rescue the effects of ZIKV-induced differentiation defects and growth in the human fetal-like forebrain organoids [99]. A recent study suggests that have been implanted cerebral organoids in lesion sites of traumatic brain injury, which differentiated into cortical neurons, generated long projections and rescued deficits in memory and learning; which will create a potential therapeutic unique method for brain injury treatment [100]. In vitro models of the blood brain barrier (BBB) is an important challenge for the study of drug development that can reach the central nervous system and BBB transport [101]. The BBB organoids have been created which represents a cost effective, versatile, and accurate, in vitro tool. BBB organoids modeling could accelerate therapeutic discovery for the treatment of several neuropathologies [102]. Cerebral organoid models probably in the soon future will be able to simulate blood flow across organs that link blood-brain barrier cultures with liver cultures, which will represent a qualitative shift in pharmacology and therapeutics in the neuroscience field. Furthermore, it would enable us to have realistic therapeutic options and realize efficacious stem cell interventions for restoration therapy or cell replacements for neurodegenerative diseases.

## 9. Advantages of Using 3D Model Compared to 2D Model in the Cerebral Investigation

- There are some conflicts between the culture of 2D, and 3D that were noted; different subtypes of neurons can be generated from OLIG2C NPCs in 3D culture, while 2D culture can only obtain CRC neurons
- The brain 3D models development has extremely contributed to neurotropic viruses' study promotion and provided alternative ZIKV infection models for 2D cell culture and animal models
- Cerebral 3D models possess important features compared to 2D classical culture, whereas the 3D models are very partially simulating the generation of pathological features for neurodegenerative diseases
- The 2D cell culture may not exactly represent modeling human diseases. Thus, using 3D models to investigate pathological and regulatory molecular mechanisms is a promising strategic choice
- Treatment of 3D models showed general therapeutic resistance with apoptotic and antiproliferative effects differing biological mechanisms from those of 2D models

## 10. Organoids Challenges

There is a high advance during the past decade in culture, generation, and using the human brain, referred to as “cerebral organoids” or “brain organoids” in the lab for research and investigation. Cerebral organoids provide a unique model to understand the evolvement of the human brain and aging progression. Up to now, cerebral organoids have been applied in researching neurological disease mechanisms, drug efficacy, etc. Researchers comprehend a few issues in the domain even though cerebral organoids possess important features compared to 2D classical culture. First, the cerebral organoids are very partially simulating the generation of pathological features for neurodegenerative diseases. Second, it is still a great challenge to mimic well the complexity of the human brain during brain development and aging, in a spatiotemporal pattern, such as the cross transmission between different cells, maturity, structure, dynamic cellular composition, etc. Third, until now, the cerebral organoids do not mimic the human tissues in typical environments like the body; particularly, the brain tumor must be in a special microenvironment that is immune suppressive. Fourth, due to the cultural methods still do not meet the need, one chamber could have some variations among organoids. This variation in the volume and the size between the patient-derived organoids and control absolutely will affect the results. Fifth, functional vasculature generation in organoids is an important challenge which is not yet been achieved; thus, this organoid technology application will require the possibility of functional vasculature generation in the future. Sixth, to culture and generate cerebral organoids, it required multiple reagents and is technically challenging. Additionally, more challenging for healthy organoids to get if culture time increases. Seventh, there will be a need in the future to rediscuss some ethical issues concerning the use of reprogrammed human cells iPSC derived and the complex brain organoid generation that will smooth the way for decreasing dramatically animal use for experiments, especially in drug discovery investigations. Hence, technical advances and more research can decrease challenges and resolve these issues in the future. Furthermore, spatial profiling, single-cell transcriptomics, and therapeutics will be major fields for research in the soon future. Finally, the establishment of unified guidelines as a catalog for human organoids that include an atlas and cultural techniques for organoids could be a great and valuable help in improving and developing medical research.

## Figures and Tables

**Table 1:** Summary for current applications of cerebral organoids modeling and their cell/tissue types.

Organoid type	Cells or tissue	Disease modelled	Characteristics/phenotype	Reference
Oligocortical spheroids	Oligodendrocyte	Hypoxic injury	OLIG2, MBP/CNP	[16, 21, 22]
Cortical spheroid	Glutamatergic neurons	Rett's syndrome	No embedding in the extracellular matrix. Generated repetitive action potentials at depolarization. Contains nonreactive astrocytes. vGLUT	[21, 23, 24]
	Neurons, neuronal progenitors	Tuberous sclerosis complex	mTORC1 hyperactivation, glia, and neuron hypertrophy.	[25]
Midbrain	Dopaminergic neurons	PD	TH, FOXA2	[4, 26, 27]
Hypothalamus or arcuate	Striatal neurons, GABAergic interneurons, hypothalamic neurons	Prader-Willi's syndrome	OTP, POMC, Rax1	[26, 28, 29]
Retinal	Optic vesicle-like structures, multizone ocular progenitor cells	Retinitis pigmentosa, end-stage AMD	CRX, OTX2, the surface filled with collagen matrix; the apical edge has dense projections like crystalline	[30–32]
Ventral forebrain	GABAergic/glutamatergic neurons	ASD, epilepsy	Interneurons integrate into a synaptically connected microphysiological system, GAD67, GABA	[21, 33]
Forebrain	NPCs	ZIKV	ZIKV-induced cell apoptosis increased, neuronal cell-layer thickness decreased, and larger ventricular lumen in small size organoids.	[34, 35]
	vRGCs	Miller-Dieker's syndrome	Reduced organoids size, typical genes of neurons and RGCs increased, atypical vRGCs cell division, cortical niche deformation with neuroepithelial loops decreasing, LHX2, EMX2, and FOXG1	[36, 37]
Dorsal forebrain	NPCs	Cytomegalovirus-induced microcephaly	Situated at the ventricular zone, induced-cystic and vacuolar degeneration, lamination necrosis of the malformed cortical, cellular proliferation dropped.	[38–40]
Forebrain assembloids	Cortical interneurons	Timothy syndrome	Integrate into cortical microcircuit and migrate in a saltatory manner. GSX2, CHAT, SP8	[19, 33, 41]
Organoid-grown microglia/dorsal forebrain	Microglia	Schizophrenia, amyotrophic lateral sclerosis	Contain astrocytes and neuronal architecture, TGFB1, CSF1, IL34, and IBA-1	[42, 43]
Neurosphere	Neurons	Herpes simplex virus (HSV)	Neurons have a vulnerability to destruction by HSV-1 lytic infection	[44]
Asteroids	Astrocyte	Amyotrophic lateral sclerosis	Transplanted into the nonimmunosuppressed mouse brain. GFAP	[8, 45, 46]
